# Ethnotaxonomical considerations and usage of ichthyofauna in a fishing community in Ceará State, Northeast Brazil

**DOI:** 10.1186/1746-4269-9-17

**Published:** 2013-03-08

**Authors:** Márcia Freire Pinto, José da Silva Mourão, Rômulo Romeu Nóbrega Alves

**Affiliations:** 1 Pós-Graduação em Etnobiologia e Conservação da Natureza, Universidade Federal Rural de Pernambuco; Pós-Graduação em Desenvolvimento e Meio Ambiente, Universidade Federal da Paraíba, João Pessoa, Brazil; 2 Department of Biology, Universidade Estadual da Paraíba, Av. das Baraúnas, s/n, Bodocongó, Campina Grande, PB, 58109-753, Brazil

**Keywords:** Folk classification, Ethnozoology, Conservation

## Abstract

**Background:**

Artisanal fishery is one of the most important economic activities for human populations living in coastal areas. The traditional knowledge that fishermen have of fishes is of utmost importance for the establishment of conservation strategies for many species. This study aimed to analyse the knowledge of and utilization of fishes by the artisanal fishermen in a fishing community on the coast of Ceará State (Northeast Brazil).

**Methods:**

In 2011, a number of semi-structured interviews were performed with fishermen with more than 20 years of fishery experience. The interviews were about fisheries (collecting spots, artefacts, etc.) and fish use. The fishes cited by the fishermen were identified scientifically and ethnotaxonomically.

**Results:**

Considered masters of fishery, they cited 162 vernacular names of fishes, which corresponded to 290 different species, also including other animals such as dolphins, porpoises, whales and manatees. The criteria for the classification of the fishes were well known and utilised by the fishermen, and they were based on morphology, behaviour, habitat and the importance of commercial and fishing activities. Four hierarchical categories were identified in their classification system: kingdom, life-form, generic and specific. The fish nomenclature created by the fishermen was mostly composed of generic and monotypic names. The main uses of fish were for food and commercial purposes.

**Conclusions:**

The results stress the richness and complexity of the knowledge of the artisanal fishermen of Redonda Beach, and they provide support for the possibility of future studies and for the development of management plans and the management of wildlife resources.

## Background

Since the dawn of societies, human beings have appropriated natural resources in search of food, energy and raw material for their activities. The diversity of ecosystems and the abundance of natural resources in coastal areas have attracted human groups since remote ages [1-3]. This fact can be proved by the existence of shell-mounds, which according to Simões [4] illustrate the food resources that the ‘primitive’ inhabitants of the coastland used for their subsistence: oysters, mussels, crabs and fishes, besides reptiles, mammals and birds.

Brazil has one of the longest coastlines in the world, with nearly 4,655 miles. This area covers a variety of ecosystems such as estuaries, mangroves, everglades, rocky shores, sandbanks, beaches, dunes, reefs, seagrass and cliffs [5]. These sites shelter a great diversity of animals utilised by the human population living along the coastal areas [1,2]. The exploitation of these resources characterises one of the most important activities of subsistence in the coastal area, artisanal fishery, which generates employment and provides food for many fishing communities. Such activity is responsible for 52.5% of the marine and estuarine fishery resources in Brazil [6]. The Northeast region is responsible for the largest share of the country’s production [6]. There are over 600 thousand Brazilian fishermen who, by working in the capture of fish and seafood and in the processing and marketing of fish, support their families and generate income for the country [7].

In recent years, a drastic decrease in artisanal fishing production has occurred. As a consequence, there has been a gradual deterioration in the life patterns of those who depend on this activity, which is influenced by biological, sociological, economic, political and institutional factors. To understand this multidimensional context, it will be necessary to come up with effective solutions to sustain the exploited species, provide cultural support and help local communities survive. Such information may be obtained through the study of ethnoichthyology, a branch of ethnozoology that aims to understand the interaction between humans and fishes, with special regard to perception, knowledge and use within each social system of the fishing communities [8,9]. The investigations carried out by the artisanal fishermen are important to establish marine ecosystem conservational measures and for the development of fishing.

The first studies of Brazil’s artisanal fishermen from an ethnobiological perspective were carried out by Forman [10,11], Cordell [12] and Maranhão [13]. There were advances in such studies after 1990, when studies were carried out with the aim of understanding the fishermen’s processes of identification, naming and classification of the animals using ethnotaxonomy (also known as folk taxonomy or popular taxonomy) and to understand how they utilise such animals [14-29].

Mourão and Montenegro [30] stress that ethnological classification studies allow an interaction between traditional populations' knowledge and scientific knowledge, which can help create an understanding of the diversity of community relations and environments. According to Garcia-Quijano and Pitchon [31], in the fishery domain, where vital resources are hard to find, local information may be important for the maintenance of viable and effective management systems. In other words, when scientific knowledge is limited, local knowledge may add to a broader perspective which is at the same time cultural and environmentally adequate.

Alves [32] states that both the sociological and the economic situation and the environmental perceptions of the local communities need to be considered in the management of fishery resources, especially those that have been overexploited. Traditional ecological knowledge has the potential to contribute to the conservation of biodiversity and to assist in the management of general ecological systems [33], because it aims to support the most different and flexible management systems [34,35], especially with regard to endangered species [36].

According to Diegues [37], a solution to the social and ecological problems of the coastal areas of Brazil is represented in local initiatives towards management. The same author highlights the fact that species are objects of knowledge and of use and domestication, can be the inspiration for myths and rituals, and finally, they have become commodities in modern societies. With regard to this, one of the goals of the present study was to produce an inventory of the fishes that are known and used by the artisanal fishermen of Ceará State (Northeast Brazil). The aim was also to record and analyse the process of how fishermen identify, name and classify the fishes. The intention was to contribute to the knowledge of the local fauna, to provide important data for the development of research, conservation of endangered species and development of management plans for marine biological resources.

## Methods

**Figure 1:**
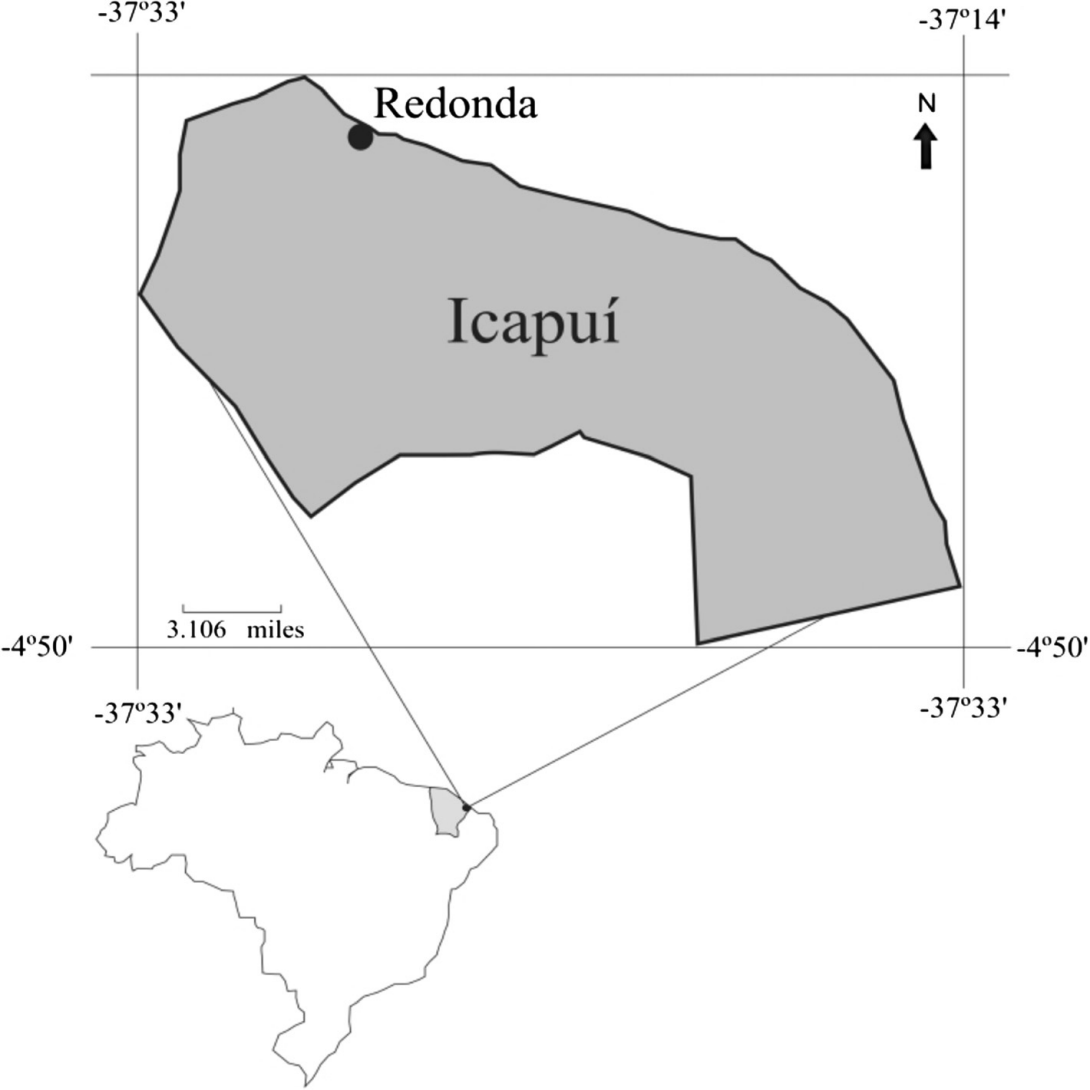
**Localization of Redonda, Icapuí County, Ceará State.** Source: adapted from Santos; Meireles & Kelting [38].

The research was carried out in 2011 with the fishermen of the fishing community in Redonda Beach (4º39’8”S and 37º27’57”W), which is located in Icapuí County, on the far eastern coast of Ceará State, in Northeast Brazil (Figure 1) [38]. The county is situated in the State Coastal Zone. It has an area of 124.7 square miles and a population of 18,572 inhabitants [39]. It is bordered on the west and the south by the city of Aracati, and on the east by Rio Grande do Norte State. Redonda Beach began to be inhabited in the late XIX century and today is one of the largest population centres of Icapuí County, with more than 3000 inhabitants.

In that region, since the 1970s, there has been a great social and environmental conflict between the fishery communities due to lobster fishing. Fishermen from neighboring communities around Redonda Beach, make illegal uses of air compressors although all of them receive closed-season insurance. Fishing is the main economic activity on the Icapuí coast. In Redonda, as in any other fishery communities of the county, lobsters (*Panulirus* spp., *Scyllarides* spp.) represent the main fishing resource although many marine animals, especially fishes, are used by communities for food and sale.

The research was approved by the Ethics Committee in Research on Humans Department of the Lauro Wanderley University Hospital, from the Federal University of Paraíba (UFPB), protocol CEP/HULW nº 662-B/10 and the permission of the syndicate of fishermen (Sindicato dos Pescadores e Marisqueiras de Icapuí) was required to interview the fishermen.

The investigation was carried out between January and December 2011. The use of fishes and fishery data was acquired by using semi-structured questions with free interviews and informal conversation. The questionnaires were given to 12 fishermen from the Redonda community who had more than 20 years of fishery experience. The community considers these men to be masters of fishery, because they have much time and experience in artisanal fishery. It is believed that the fishermen with over 20 years of fishing experience hold greater knowledge about fishery resources, regardless of age. The questionnaire investigated each fishery (collecting spots, artefacts, etc.) and fish uses.

The questionnaires utilised the technique of the Free List [40,41] by which the fishermen freely listed the fishes they knew and used. To complement this, the following methods were adopted: Nonspecific prompting, in which the informant was asked whenever he said that he did not remember any other item, and New reading, whereby the researcher reads every item cited by the informant to refresh his memory [42].

The fishes cited by the fishermen were identified scientifically and ethnotaxonomically. In terms of ethnotaxonomic dentification, the popular names of fishes and some of their characteristics were listed so that the fishermen could classify and cluster them. On the basis of the scientific identification of each taxonomic group, pictures were presented illustrating each animal so that the fishermen could confirm their identification of the animals [43,44]. The data were classified by using the principles of categorization and nomenclature proposed by Berlin [45] and Berlin, Breedlove and Raven [46]. In such forms of classification, the living beings are gathered in the following hierarchical levels, according to the fishermen’s knowledge: Kingdom (unique beginner), Life-form, Generic and Specific.

The species of fishes were identified from photographs and previous research was done near the study area [47], from Northeast Brazil [48-50] and from the Fish Base database (http://www.fishbase.org). For further analyses marine animals, also considered as fishes by the informants, the data bank of the studies performed by AQUASIS (Association for Aquatic Ecosystems Studies and Preservation) at Icapuí and the assistance of professionals from the Federal University of Ceará were used.

## Results and discussion

### Ethnotaxonomical considerations

Following Berlinian principles, the animals were considered to be in the first hierarchical level of the folk classification, the Animal Kingdom. While the Life-form, the second hierarchical level of the folk classification system, was established by being mainly based on habitat and body shape, the fishermen clustered the animals as “peixes” (fishes), “crustáceos” (crustaceans), “mariscos” (shellfish) and “tartarugas” (turtles).

The categories used by the fishermen were based on standards related to their morphology, habitat, importance in commerce and the kind of fishery. It was stated that the criteria for the classification of living beings used by local fishermen extrapolated the morphological limits of species, as proposed by Berlin [45], Hunn [51] and Brown [52], whereby morphological characters are not the only standards of classifying living beings, although they are very important.

The fishermen clustered all of fishes, whales, dolphins, porpoises and manatees in the ‘fish’ group (life-form). This corroborates a tendency recorded in the studies of Mourão and Nordi [24] that fishermen gather, in a flexible way, some aquatic mammals and invertebrates into the ‘fish’ category because of the resemblance of their bodies to fish and because they belong to the same habitat. The flexibility of the ‘fish’ category was also recognised by Marques [14], Paz and Begossi [18] and Costa-Neto [53] among the fishermen they studied.

It is known that the term ‘fish’ is utilised nowadays for convenience and not as a taxonomic unit, since fish are not a monophyletic group. In terms of systematic phylogenetic categorisation, the fishes belong to six independent evolutionary lineages [54]. One of them is the Chondrichthyes. Sharks and stingrays belong to the class Chondrichthyes and the subclass Elasmobranchii [55-57], which includes fishes with cartilaginous skeletons. At the study site, the fishermen distinguished shark or dogfish and stingrays from the other fishes, despite all of them being classified as ‘fish’ in terms of life-forms. The criteria used by fishermen in making this distinction were morphological characters such as size, form and presence of fin, body shape, and presence and type of scales and teeth.

**Figure 2:**
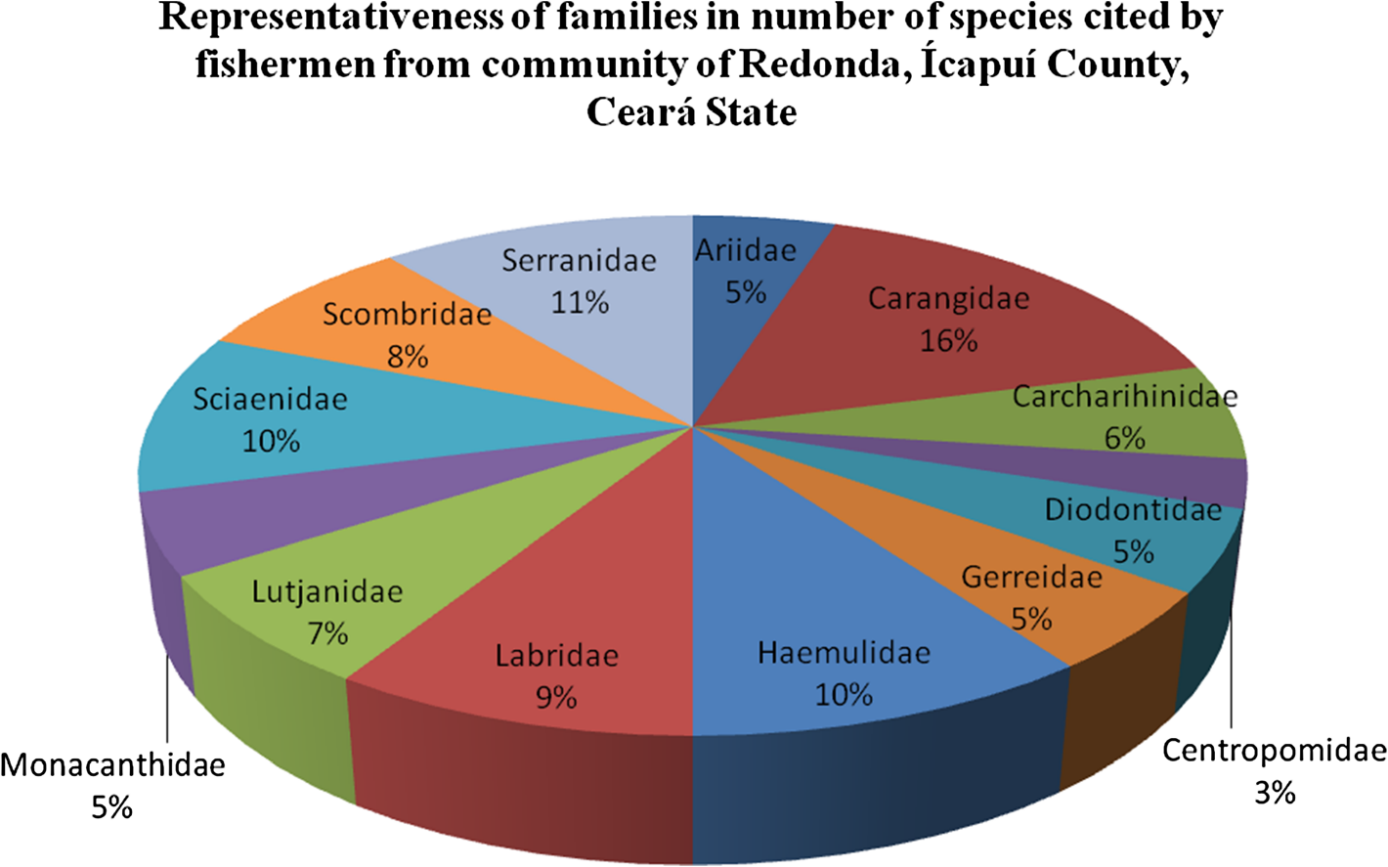
Percentage distribution of main fish families cited by fishermen of Redonda Beach, Ceará State.

The fishermen cited 162 fish names that corresponded to 281 fish scientific species (Additional file 1). The fish species were distributed in 72 taxonomic families, some highlighted by the majority of citations (Carangidae, Serranidae, Haemulidae and Scianidae), by food and commercial use (Labridae, Scombridae, Lutjanidae, Monacanthidae, Ariidae, Diodontidae, Gerreidae and Centropomidae) and some that were classified as endangered species (Carchahinidae and Lutjanidae) (Figure 2).

The mammals that were considered by the fishermen to be fishes represented only 3% of the total animals cited. The fishermen mentioned manatees (*Trichechus manatus manatus*), whales (*Physeter macrocephalus, Globicephala macrorhynchus* and *Megaptera novaeangliae*), dolphins (*Stenella frontalis, Stenella clymene, Stenella coeruleoalba* and *Steno bredanensis,*) and porpoises (*Sotalia guianensis*) as ‘fishes’ that were not used but were easy to identify because they could be observed within the study area.

The majority of fishes cited by the fishermen were at the generic ethnotaxonomic level. This corroborated the proposition of Berlin [45] that the generics will be the majority in all of the folk systems. According to Berlin [45], the generic taxonomy may be arranged in two ways: generic monotypic, when the generic does not possess a lower category, and generic polytypic, when it is divided in a specific way.

The fishes that were mentioned by the fishermen in a more detailed and binominal way were considered folk specific and had a commercial and/or cultural importance. An example of a generic polytypic animal that was not utilised for food or commercial purposes was the dolphin (considered a fish by fishermen), which was subdivided into ‘spotted dolphins’ and ‘white dolphins’, showing that a cultural relationship existed between the dolphins and the fishermen.

Berlin [45] defends the intellectual or idealistic way of thinking by which humans, anywhere of the world, are capable of recognising the structure and inherent order of the biological world, independent of the practical value of animals and plants. However, among the criteria used by fishermen to identify, name and classify fishes it is noticed that the character utility is much employed. In this study, the fishermen identified and named several fishes that could be classified under the specific folk level. Most of time it was possible to see that the fishermen utilized morphological criteria (colouration, body shape, type of mouth, thorn presence, type of fin, head shape), habitat criteria (deep, substratum), behaviour (trembling, shock) and criteria for making an analogy with other animals, objects or even with local people in the community (Table 1). This same tendency was documented by Begossi and Garavello [58], Marques [14] and Mourão and Nordi [24].

**Table 1:** Characters used by artisanal fishermen of Redonda Beach to identify and name some fishes

**Characters used by fishermen to identify and name fishes**		
**Characters**	**Vernacular name**	**Scientific name**
**Morphologicals**		
Coloration	Parum-branco	*Chaetodipterus faber* (Broussonet, 1782)
Body shape	Zambai-roliça	*Tylosurus crocodilus crocodilus* (Péron & Lesueur, 1821)
Type of mouth	Bagre-beiçudo	*Notarius grandicassis* (Valenciennes, 1840)
	Boca-mole	*Larimus breviceps* (Cuvier, 1830)
Presence of spines	Baiacu-espinho	*Diodon* spp. */ Chilomycterus* spp.
Type of fin	Bagre-de-fita	*Bagre bagre* (Linnaeus, 1766)
Head shape	Tubarão-cabeça-de-martelo	*Sphyrna tiburo* (Linnaeus, 1758)
		*Sphyrna lewini* (Griffith & Smith, 1834)
		*Sphyrna zygaena* (Linnaeus, 1758)
**Type of habitat**		
Depth	Bagre-da-costa	*Aspistor luniscutis* (Valenciennes, 1840)
Substratum	Arraia-de-pedra	*Dasyatis* spp.
**Behaviour**		
	Cação-choque (arraia-choque)	*Narcine* spp.
**Analogy with other animals**		
	Zambaia-cachorro	*Strongylura timucu* (Walbaum, 1792)
	Cação-jaguara	*Galeocerdo cuvier* (Péron & LeSueur, 1822)
	Peixe-morcego	*Ogcocephalus vespertilio* (Linnaeus, 1758)
	Baiacu-vaquinha	*Acanthostracion polygonius* (Poey, 1876)
	Tubarão-baleia	*Rhincodon typus* (Smith, 1828)
	Cavalo-marinho	*Hippocampus* spp.
**Analogy with objects**		
	Agulhão-de-vela	*Xiphias gladius* (Linnaeus, 1758)
	Arraia-bico-de-remo	*Dasyatis guttata* (Bloch & Schneider, 1801)
	Cação-toalha	*Mustelus canis* (Mitchill, 1815)
	Carro-de-boi	*Anisotremus virginicus* (Linnaeus, 1758)
	Cação-bola	*Carcharhinus signatus* (Poey, 1868)
**Analogy with people**		
	Baiacu-araldo	*Lactophrys trigonus* (Linnaeus, 1758)
	Baiacu-carlitão	*Lactophrys* sp.

The generic polytypic “bagre” (catfish) was cited by the largest number of fishermen as “bagre-branco” (*Genidens barbus, Sciades herzbergii*), “bagre-beiçudo” (*Notarius grandicassis*), “bagre-canhacoco”, “bagre-da-costa” and “bagre-areiaçu” *(Aspistor luniscutis, Sciades proops*), “bagre-de-fita” (*Bagre bagre, Bagre marinus*), “bagre-mandim” and “bagre-amarelo” (*Cathorops spixi*). Mourão and Nordi [24] emphasise that it is justly in the generic polytypic designation, since it was in the groups of animals with the highest economic and cultural value and psychological importance.

According to Berlin [45] it is possible to recognise through scientific comparison at least three types of correspondence: correspondence one-to-one (1:1), when a single *folk* generic taxa refers to only one scientific species; over-differentiation, when two or more *folk* generic taxa refer to a single scientific species; under-differentiation type I, when a single *folk* generic taxa refers to two or more species of the same scientific genus; and under-differentiation type II, when an single *folk* generic taxa refers to two or more species of two or more genera.

Comparing the 162 *folk* generic taxa obtained in this research with the scientific literature, it was found that 65 had one-to-one correspondence (1:1) (Table 2), 17 over-differentiation correspondence, 49 under-differentiation type I, and 38 under-differentiation type II. In some cases, folk generic taxa referred to species of the same genera as wells as different genera, for example, the “garajuba” corresponding to the following species: *Caranx ruber, Caranx crysos, Carangoides bartholomei* and *Seriola dumerili.* On the other hand, “baiacu-espinho”, which was also cited as “baiacu-espinheiro”, corresponded to different species of the same genus (*Diodon hystrix, Diodon holocanthus*) and different genera (*Chilomycterus antillarum, Cyclichthys schoepfi*).

**Table 2:** One-to-one correspondence between scientific species and folk generic taxa

**One-to-one correspondence between scientific species and folk generic taxa**	
**Family/Scientific species**	**Folk generic taxa**
**Ariidae**	
*Notarius grandicassis* (Valenciennes, 1840)	Bagre-beiçudo
**Atherinidae**	
*Atherinella brasiliensis* (Quoy & Gaimard, 1824)	Manjuba
**Belonidae**	
*Strongylura timucu* (Walbaum, 1792)	Zambaia-cachorro
**Batrachoididae**	
*Thalassophryne nattereri (Steindachner, 1876)*	Aniquim; Anequim
**Carangidae**	
*Elagatis bipinnulata* (Quoy & Gaimard, 1825)	Arabaiana
*Trachinotus falcatus* (Linnaeus, 1758)	Garabebéu
*Trachinotus carolinus* (Linnaeus, 1766)	Pampo-amarelo
*Trachinotus goodei* (Jordan & Evermann, 1896)	Pampo-branco
*Chloroscombrus chrysurus (Linnaeus, 1766)*	Pelombeta; palombeta
*Caranx hippos* (Linnaeus, 1766)	Xaréu
**Carcharhinidae**	
*Galeocerdo cuvier* (Péron & LeSueur, 1822)	Cação-jaguara
*Carcharhinus porosus* (Ranzani, 1839)	Cação-lombo-preto
**Chaetodontidae**	
*Chaetodon striatus* (Linnaeus, 1758)	Parum-dourado
**Clupeidae**	
*Chirocentrodon bleekerianus (Poey, 1867)*	Arenque; Arem; Erem
**Dasyatidae**	
*Dasyatis marianae* (Gomes, Rosa & Gadig, 2000)	Arraia-coã
**Elopidae**	
*Elops saurus* (Linnaeus, 1766)	Ubarana-espinhenta
**Ephippidae**	
*Chaetodipterus faber* (Broussonet, 1782)	Parum-branco
**Fistulariidae**	
*Fistularia tabacaria* (Linnaeus, 1758)	Trombeta
**Ginglymostomatidae**	
*Ginglymostoma cirratum* (Bonnaterre, 1788)	Tubarão-lixa
**Gymnuridae**	
*Gymnura micrura* (Bloch & Schneider, 1801)	Arraia-jamanta
*Gymnura altavela* (Linnaeus, 1758)	Arraia-lisa
**Haemulidae**	
*Orthopristis ruber* (*Cuvier, 1830*)	Canguite; canguito; quanguite
*Anisotremus virginicus (Linnaeus, 1758)*	Carro-de-boi; Boi-de-carro
*Pomadasys corvinaeformis* (Steindachner, 1868)	Coró-branco
*Conodon nobilis* (Linnaeus, 1758)	Coró-cardeiro
*Haemulon flavolineatum* (Desmarest, 1823)	Listrado
*Anisotremus surinamensis* (Bloch, 1791)	Pirambú
*Haemulon melanurum* (Linnaeus, 1758)	Sapuruna-de-listras
**Hemiramphidae**	
*Hyporhamphus unifasciatus* (Ranzani, 1842)	Agulha-branca
**Holocentridae**	
*Holocentrus adscensionis* (Osbeck, 1765)	Mariquita
**Kyphosidae**	
*Kyphosus incisor* (Cuvier, 1831)	Salema-azul
**Labridae**	
*Caulolatilus chrysops* (Valenciennes, 1833)	Batata
**Lamnidae**	
*Carcharodon carcharias* (Linnaeus, 1758)	Tubarão-branco
***Lobotidae***	
*Lobotes surinamensis* (Bloch, 1790)	Xacarona; xancarrona
**Lutjanidae**	
*Lutjanus synagris* (Linnaeus, 1758)	Ariacó
*Lutjanus apodus* (Walbaum, 1792)	Carapitanga
*Lutjanus analis* (Cuvier, 1828)	Cioba
*Lutjanus jocu* (Bloch & Schneider, 1801)	Dentão
*Lutjanus vivanus* (Cuvier, 1828)	Pargo-vidrado; Pargo-vridado
**Malacanthidae**	
*Malacanthus plumieri* (Bloch, 1786)	Pirá
**Megalopidae**	
*Megalops atlanticus* (Valenciennes, 1847)	Camurim
**Mobulidae**	
*Manta birostris* (Walbaum, 1792)	Arraia-de-orelha
**Myliobatidae**	
*Aetobatus narinari* (Euphrasen, 1790)	Arraia-pintada
**Ogcocephalidae**	
*Ogcocephalus vespertilio* (Linnaeus, 1758)	Peixe-morcego
**Polynemidae**	
*Polydactylus virginicus* (Linnaeus, 1758)	Barbado; barbudo
**Pomacanthidae**	
*Pomacanthus paru* (Bloch, 1787)	Parum-preto
**Priacanthidae**	
*Selar crumenophthalmus* (Bloch, 1793)	Garapau
**Sciaenidae**	
*Larimus breviceps* (Cuvier, 1830)	Boca-mole
*Cynoscion leiarchus* (Cuvier, 1830)	Pescada-branca
*Macrodon ancylodon* (Bloch & Schneider, 1801)	Pescada-curuvina
*Cynoscion* sp.	Pescada-amarela
*Cynoscion* sp.	Pescada-bico-fino
*Cynoscion* sp.	Pescada-ticupá
**Scombridae**	
*Aconthocybium solandri* (Cuvier, 1832)	Cavala
**Scorpaenidae**	
*Scorpaena plumieri* (Bloch, 1789)	Aniquim; anequim
**Serranidae**	
*Epinephelus adscensionis* (Osbeck, 1765)	Gato
*Epinephelus itajara* (Lichtenstein, 1822)	Mero
*Myripristis jacobus* (Cuvier, 1829)	Oiuda
*Etelis oculatus* (Valenciennes, 1828)	Pargo-piranga
*Serranus flaviventris* (Cuvier, 1829)	Sapé
**Sparidae**	
*Pagrus pagrus* (Linnaeus, 1758)	Salema
**Sphyraenidae**	
*Sphyraena barracuda* (Walbaum, 1792)	Barracuda
**Stromateidae**	
*Peprilus paru* (Linnaeus, 1758)	Mocinha
**Tetraodontidae**	
*Lactophrys trigonus* (Linnaeus, 1758)	Baiacu-araldo
*Lactophrys* sp.	Baiacu-carlitão

In the research of Seixas and Begossi [59], in São Paulo, the authors identified another kind of correspondence, called over-differentiation type II, where two or more folk generic taxa correspond to two or more scientific species. This type of correspondence was also observed in four cases in the present study: “bagre-canhacoco”, “bagre-da-costa” and “bagre-areiaçu” (*Aspistor luniscutis, Sciades proops*), “baiacu-de-chifre” or “baiacu-vaquinha” (*Acanthostracion quadricornis, Acanthostracion polygonius*); “cação-choque” or “choqueiro” (*Narcine bancrofti, Narcine brasiliensis*), and “cação-panã”, “tubarão-cornuda”, “martelo”, “tubarão-cabeça-de-martelo” and “tintureira” (*Sphyrna tiburo, Sphyrna lewini, Sphyrna zygaena*). Such cases show that the correlations proposed by Berlin [45] may overlap.

According to Berlin [45], the one-to-one correspondence (1:1) may be evidence of the diversity of organisms existing in a community, demonstrating that the local population possesses detailed knowledge about several aspects of the living organisms that they classify. Over-differentiation tends to generally occur with organisms that are culturally significant for utilitarian or cognitive reasons [60].

The major correspondence obtained in this study was the one-to-one (1:1), which was also observed by Seixas and Begossi [59]. Notwithstanding, this correspondence was not observed by Clauzet et al. [61] in their study with the fishermen of Guaibim Beach, Bahia.

High correspondence emphasizes the importance of considering the local fishermen’s knowledge and accumulated experience in biological inventories [62], besides contributing to the development of management plans for fishery resources [43,63,64].

Besides the nomenclature based on animals’ morphological characters, another recurrent standard folk nomenclature is ontogeny, which is represented by fishermen as different sizes of individuals of the same species [24]. The fisherman of Redonda’s beach cited “tainha” (mullet) and “pema” (generic monotypic) as “sauna” (*Mugil* spp.) and “camurupim” (*Centropomus* spp.) puppies, respectively. In their ethnotaxonomic studies, Seixas and Begossi [59], Costa-Neto and Marques [19], and Mourão and Nordi [24] reported the importance of the ontogenic criteria of nomenclature.

### Fish utilization according to fishermen

Fishes have a great importance for food in the fishing community at Redonda Beach, although many of them are also sold by fishermen to other Brazilian regions and even to other countries. In such case, the exported species are, for example, the “serra” (sawfish) (*Scomberomorus cavalla, S. brasiliensis,S. regalis*) and “albacora" (*Euthynnus alletteratus, Thunnus alalunga, T. albacores, T. atlanticus*).

The fishes (and other animals considered to be fishes by the fishermen) were cited mostly according to their food uses. Some species, however, were mentioned for other reasons: a) they represent danger ^a^, such as “aniquim” (*Thalassophryne nattereri*, *Scorpaena plumieri*)*,* “tubarão-branco” (white shark) (*Carcharodon carcharias*), “choqueiro” (*Narcine bancrofti*, *N. brasiliensis*), “baiacu-de-chifre” (scrawled cowfish) (*Acanthostracion quadricornis*, *A. polygonius*) and “tubarão-cabeça-de-martelo” (hammerhead) (*Sphyrna tiburo, S. lewini, S. zygaena*); b) they provoke repulsion because of their body shape, such as “peixe-morcego” (batfish) (*Ogcocephalus vespertilio*), “piolho” (*Echeneis naucrates*) and “muriongo” (*Myrichthys ocellatus*; *Myrichthys breviceps*, *Myrophis punctatus*); c) their meat has a bad taste, such as “avuador-de-casco” (*Cheilopogon melanurus*, *C. cyanopterus*, *Exocoetus volitans*) and “arraia-pintada” (*Aetobatus narinari*); d) they possess little meat, such as “trombeta” (*Fistularia tabacaria*), “mariquita” (*Holocentrus adscensionis*), “solha” (*Achirus achirus*, *A. lineatus*, *Trinectes paulistanus*, *Citharichthys macrops, C. spilopterus*, *Bothus lunatus*, *B. ocellatus*, *B. robinsi*, *Cyclopsetta fimbriata*, *Symphurus diomedianus*, *Etropus crossotus*, *Paralichthys brasiliensis*, *Syacium micrurum*, *S. papillosum*), “cação-viola” (*Rhinobatos percellens*, *R. lentiginosus*) and “cavalo-marinho” (seahorse) (*Hippocampus reidi*, *H. aff. erectus*) or e) are very large, such as “baleias” (whales) (*Megaptera novaeangliae*, *Globicephala macrorhynchus*, *Physeter macrocephalus*) and the “tubarão-baleia” (*Rhincodon typus*).

The multiple uses of fishes and their interactions with fishermen in fishing motivate preferences and disinclinations. As pointed out by Hanazaki and Begossi [65], this could be explained by ecological and cultural factors, in other words, by the resources that are available, by the species’ position in the food chain and by the importance of those species in the economy and in their community’s social relations.

Of the total of 281 fish scientific species identified, 62 were exclusively used for food, 188 were sold and utilized for local consumption, two were used for handicrafts (which were also sold), and 29 species were not utilized. The main use of fishes was for sale, with emphasis on the “sirigado” species (*Mycteropercs bonsci, Paralabrax dewegeri*), “cavala” (mackerel) (*Aconthocybium solandri*), camurupim (*Centropomus* spp.), “ariacó” (*Lutjanus synagris*), “carapicu” (*Eucinostomus* spp.) and “tibiro” (*Oligoplites* spp.), which were most often cited by the fishermen interviewed.

The “baiacu-espinho”, a name that could refer to several species (*Diodon hystrix, Diodon holocanthus, Chilomycterus antillarum, Chilomycterus antennatus, Chilomycterus atringa, Cyclichthys schoepfi, Chilomycterus spinosus*), was among the fishes most cited by the fishermen used exclusively for bait, because according to them, this fish is poisonous and nobody wants to buy it. The lack of interest in buying or selling “baiacu” has also been reported by other researchers in Brazil [66,67]. This rejection is related to the toxicity of two neurotoxin (tetrodontoxin and saxitoxin) found in some puffers and porcupine fishes [68,69].

All parts of fishes are sold, with the exception of some sharks in which only the ‘aba’ (fins) are sold for US$ 205.00 per kilogram. In this case, according to fishermen, this trade is carried out by people from other localities. The fishermen noted that the ‘aba’ is sold to merchants in the city of Fortaleza, and is then exported to Asian countries. This indicates that shark fishing in this region is not only for meat consumption but also the sale of the fins.

The fishermen also stated that in the past marine manatees (*Trichechus manatus manatus*) served as food, although it was very difficult to find them in Redonda waters. The only fish cited by the fishermen for craftwork (earrings, necklace and decorative objects) was the seahorse (*Hippocampus reidi*). This use, called zoohandicrafts by Alves *et al*. [70], consists of any type of craftwork that utilizes animals or parts of animals, as a form of typical artistic and cultural expression, also used in other places in Brazil [71].

#### Implications of conservation

Data from FAO [72] reveal that world fishery stocks are declining and that the majority of the ten most important species of fish (which represent 30% of the production of world fisheries) are completely exploited. Thus, for fish to supply human needs, conservational measures are needed aimed at fish protection and also the sustainability of the fishing community.

There are several factors that threaten marine ichthyofauna, such as the lack of management of marine resources, which may lead to i) overfishing and bycatch, ii) the use and occupation of the coast in a disorganised way with domestic sewage, iii) the siltation of coastal basins, iv) the destruction of mangroves and other associated estuarine vegetation, v) oil spills, vi) and submarine vegetation destruction and the substratum alteration caused by bottom trawling, especially shrimp trawling [73]. In Brazil, besides these factors, there are also problems related to illegal fishing in protected areas, which involves nets with inappropriate dimensions and meshes and the use of explosives. Rosa and Menezes [74] stress that knowledge of the current state of conservation of Brazilian fish species is merely incipient. In view of this scenario, the establishment of efficient conservation measures requires an understanding of the cultural social context associated with the use of marine biological resources, highlighting the importance of ethnozoological studies

According to the Ministry of Environment [75], 19 fish species are listed as being endangered in Ceará State, of which 5 (*Carcharhinus signatus, Carcharhinus porosus, Ginglymostoma cirratum, Lutjanus analis* and *Rhincodon typus*) were mentioned by Redonda’s fishermen. The manatee, also considered a fish by fishermen, is currently the most endangered marine mammal in Brazil [76] and is on the list of endangered animals of IBAMA [77] and of the IUCN [78].

In Brazil, although manatees have been protected by law since 1967 (Lei de Proteção à Fauna No. 5.197/1967), it was only in 1980 that concerns about their conservation became publicised through the creation of the “Projeto Peixe-boi Marinho” of IBAMA [79]. With the conservation actions of non-governmental organizations in Icapuí County, Redonda’s fishermen have stopped fishing these animals.

From a conservational perspective, shark fishing deserves mention, since it is directed at removing fins and discarding the carcass, which is thrown back into the sea. This is an illegal practice called finning. Shark fishing which is carried out exclusively to obtain fins is forbidden in Brazil by the ordinance of IBAMA No. 121, de 24 de Agosto de 1998. Rose [80] asserts that, in spite of being forbidden, finning remains a highly profitable practice due to the high value of fins, which may vary from US$ 50.00 to US$ 500.00 per kilogram in oriental markets where fins are considered to be a delicacy. Finning causes the death of millions of sharks, some rare and vulnerable, directly affecting the marine ecosystem in which sharks are predators [81].

Also worth mentioning is the use of seahorses (*Hippocampus reidi*), cited by the fishermen, although they are not on the list of endangered species of IUCN; they are one of the ornamental products found along all Brazilian coasts and inland waterways [71,82]. They are used for medicine, magic, religion and ornamentation [82,83]. The inclusion of seahorses in Appendix II of the CITES convention, to which Brazil is a signatory, implies that trade in these animals must be controlled to avoid their use, which is incompatible with the survival of wild populations. The inclusion of two nominal species of seahorses (*Hippocampus reidi* and *Hippocampus aff. erectus*) in the National List of the Aquatic Invertebrates and Fish Species Overexploited or Threatened of Overexploitation implies their need for attention in relation to uses and commerce, as pointed out in the proposal of management plans for seahorse species in Brazil [84].

## Conclusions

The wealth of registered fishery resources (290 species) in the study area strengthens the socio-economic and cultural role of these animals with regard to the local coastal communities. Within the study area, the fishery resources are explored for different reasons, especially as source of food and income. The fishermen possess a vast knowledge of the biology of the species and their ecology, which is reflected in the ethnotaxonomic standards used to name, to identify and to classify them. The results show that populations involved with fishing may provide important information for scientific studies and can also contribute to the establishment of conservationist management practices and measures that aim to preserve biological diversity and cultural development. Traditional knowledge of the use of natural resources must be considered an important source of information about the current status of the resources, particularly the most fished species, the environmental impact on fisheries, the dynamics of ecosystems and local environmental characteristics, so as to provide theoretical and practical information about how to manage, to preserve and to use the natural resources in the most sustainable way.

## Endnotes

^a^Fish that are dangerous are those that are poisonous, attack humans, give electric shock or have spines that can injure people.

## Competing interests

The authors declare that they have no competing interests.

## Authors’ contributions

MFP, JSM and RRNA – Writing of the manuscript, literature survey and interpretation; MFP and RRNA –Ethnozoological data and analysis of taxonomic aspects. All authors read and approved the final manuscript.

## Supplementary Material

Additional file 1 Fish species and their respective uses in the coast of Ceará State (Northeast Brazil). Click here for file
