# Supplementary material for: Ethnotaxonomical considerations and usage of ichthyofauna in a fishing community in Ceará State, Northeast Brazil
Source: J Ethnobiol Ethnomed. 2013 Mar 8;9:17. doi: 10.1186/1746-4269-9-17 (PMC3606429; doi:10.1186/1746-4269-9-17)
Supplement: Additional file 1 — Fish species and their respective uses in the coast of Ceará State (Northeast Brazil). [file 1746-4269-9-17-S1.docx]

Additional file 01 - Fish **species** and their respective uses in the coast of Ceará State (Northeast Brazil).

| **Family/ Species** | **Specifics folk** | **Uses** |
| --- | --- | --- |
| **Acanthuridae** | | |
| *Acanthurus bahianus* (Castelnau, 1855) | Caraúna | F; C |
| *Acanthurus chirurgus* (Bloch, 1787) | Caraúna | F; C |
| **Achiridae** |  |  |
| *Achirus achirus* (Linnaeus, 1758) | Sóia; Solha | F |
| *Achirus lineatus* (Linnaeus, 1758) | Sóia; solha | F |
| *Trinectes paulistanus* (Miranda-Ribeiro, 1915) | Sóia; solha | F |
| **Albulidae** | | |
| *Albula vulpes* (Linnaeus, 1758) | Ubarana-branca; ubarana-boca-de-rato | F; C |
| **Ariidae** | | |
| *Genidens barbus* (Lacepède, 1803) | Bagre-branco | F; C |
| *Sciades herzbergii* (Bloch, 1794) | Bagre-branco | F; C |
| *Notarius grandicassis* (Valenciennes, 1840) | Bagre-beiçudo | F; C |
| *Aspistor luniscutis* (Valenciennes, 1840) | Bagre-canhacoco; bagre-da-costa; bagre-areiaçu | F; C |
| *Sciades proops* (Valenciennes, 1840) | Bagre-canhacoco; bagre-da-costa; bagre-areiaçu | F; C |
| *Bagre bagre* (Linnaeus, 1766) | Bagre-de-fita | F; C |
| *Bagre marinus* (Mitchill, 1815) | Bagre-de-fita | F; C |
| *Cathorops spixi* (Agassiz, 1829) | Bagre-mandim; bagre-amarelo | F; C |
| **Atherinidae** | | |
| *Atherinella brasiliensis* (Quoy & Gaimard, 1824) | Manjuba | F |
| **Balistidae** |  |  |
| *Balistes vetula* (Linnaeus, 1758) | Cangulo; canguro | F; C |
| *Balistes capriscus* (Gmelin, 1788) | Cangulo; canguro | F; C |
| *Melichthys niger* (Bloch, 1786) | Cangulo; canguro | F; C |
| *Xanthichthys ringens* (Linnaeus, 1758) | Cangulo; canguro | F; C |
| **Batrachoididae** | | |
| *Thalassophryne nattereri* (Steindachner, 1876) | Aniquim; anequim | F |
| *Amphichthys cryptocentrus* (Valenciennes, 1837) | Pacamon; pacamão | F |
| *Batrachoides surinamensis* (Bloch & Schneider, 1801) | Pacamon; pacamão | F |
| **Belonidae** | | |
| *Ablennes hians* (Valenciennes, 1846) | Zambai-agulha; zambaia-agulha | F |
| *Strongylura timucu* (Walbaum, 1792) | Zambaia-cachorro | F |
| *Tylosurus crocodilus crocodilus*   (Péron & Lesueur, 1821) | Zambai-roliça; zambaia-roliça | F |
| *Strongylura marina* (Walbaum, 1792) | Zambai-roliça; zambaia-roliça | F |
| **Bothidae** | | |
| *Bothus lunatus* (Linnaeus, 1758) | Sóia; solha | F |
| *Bothus ocellatus* (Agassiz, 1831) | Sóia; solha | F |
| *Bothus robinsi* (Topp & Holf, 1972) | Sóia; solha | F |
| **Carangidae** | | |
| *Elagatis bipinnulata* (Quoy & Gaimard, 1825) | Arabaiana | F; C |
| *Trachurus lathami* (Nichols, 1920) | Chincharro; chicharro | F; C |
| *Hemicaranx amblyrhynchus* (Cuvier, 1833) | Chincharro; chicharro | F; C |
| *Selene brownii* (Cuvier, 1816) | Galo | F; C |
| *Selene vomer (*Linnaeus, 1758) | Galo | F; C |
| *Selene setapinnis* (Mitchill, 1815) | Galo | F; C |
| *Alectis ciliaris* (Bloch, 1787) | Galo | F; C |
| *Trachinotus falcatus* (Linnaeus, 1758) | Garabebéu | F; C |
| *Caranx latus* (Agassiz, 1831) | Garachimbora; guaracimbora | F; C |
| *Caranx lugubris* (Poey, 1860) | Garachimbora; guaracimbora | F; C |
| *Carangoides bartholomei* (Cuvier, 1833) | Garajuba; guarajuba | F; C |
| *Caranx ruber* (Bloch, 1793) | Garajuba; guarajuba | F; C |
| *Caranx crysos* (Mitchill, 1815) | Garajuba; guarajuba | F; C |
| *Seriola dumerili* (Risso, 1810) | Garajuba; guarajuba | F; C |
| *Elagatis bipinnulata* (Quoy & Gaimard, 1825) | Guaxumba; guaxuma | F; C |
| *Seriola lalandi* (Valenciennes, 1833) | Guaxumba; guaxuma | F; C |
| *Trachurus lathami*(Nichols, 1920) | Oiam; olhão | F; C |
| *Trachinotus carolinus* (Linnaeus, 1766) | Pampo-amarelo | F; C |
| *Trachinotus goodei* (Jordan & Evermann, 1896) | Pampo-branco | F; C |
| *Chloroscombrus chrysurus* (Linnaeus, 1766) | Pelombeta; palombeta | F; C |
| *Oligoplites saliens* (Bloch, 1793) | Tibiro | F; C |
| *Oligoplites saurus* (Bloch & Schneider, 1801) | Tibiro | F; C |
| *Oligoplites palometa* (Cuvier, 1832) | Tibiro | F; C |
| *Caranx hippos* (Linnaeus, 1766) | Xaréu | F; C |
| **Carcharhinidae** | | |
| *Carcharhinus signatus* (Poey, 1868) | Cação-bola; cação-curilobola | F; C |
| *Carcharhinus acronotus* (Poey, 1861) | Cação-de-couro; Cação-flamengo | F; C |
| *Galeocerdo cuvier* (Péron & LeSueur, 1822) | Cação-jaguara | F; C |
| *Carcharhinus porosus* (Ranzani, 1839) | Cação-lombo-preto | F; C |
| *Carcharhinus falciformis* (Bibron, 1839) | Cação-rabo-seco | F; C |
| *Rhizoprionodon lalandii* (Valenciennes, 1839) | Cação-rabo-seco | F; C |
| *Rhizoprionodon porosus* (Poey, 1861) | Cação-rabo-seco | F; C |
| *Carcharhinus limbatus* (Valenciennes, 1839) | Cação-sicurí | F; C |
| *Carcharhinus acronotus* (Poey, 1860) | Cação-sicurí | F; C |
| **Centropomidae** | | |
| *Centropomus undecimallis* (Bloch, 1792) | Camurupim | F; C |
| *Centropomus parallellus* (Poey, 1860) | Camurupim | F; C |
| *Centropomus ensiferus* (Poey, 1860) | Camurupim | F; C |
| *Cetropomus undecimalis* (Bloch, 1792) | Camurupim | F; C |
| *Centropomus pectinatus* (Poey, 1860) | Camurupim | F; C |
| **Chaetodontidae** | | |
| *Chaetodon striatus* (Linnaeus, 1758) | Parum-dourado | F; C |
| **Clupeidae** | | |
| *Chirocentrodon bleekerianus (Poey, 1867)* | Arenque; Arem; Erem | F; C |
| *Harengula clupeola* (Cuvier, 1829) | Sardinha | F; C |
| *Sardinella brasiliensis* (Steindachner, 1879) | Sardinha | F; C |
| *Platanichthys platana* (Regan, 1917) | Sardinha | F; C |
| **Coryphaenidae** | | |
| *Coryphaena hippurus* (Linnaeus, 1758) | Dourado | F; C |
| *Coryphaena equiselis* (Linnaeus, 1758) | Dourado | F; C |
| **Cynoglosidae** | | |
| *Cyclopsetta fimbriata* (Goode & Bean, 1885) | Sóia; solha | F |
| *Symphurus diomedianus* (Goode & Bean, 1885) | Sóia; solha | F |
| **Dasyatidae** |  |  |
| *Dasyatis guttata* (Bloch & Schneider, 1801) | Arraia-bico-de-remo; arraia-lixa | F; C |
| *Dasyatis marianae* (Gomes, Rosa & Gadig, 2000) | Arraia-coã | F; C |
| *Dasyatis americana (Hilbebrand & Schroeder, 1928)* | Arraia-de-pedra | F; C |
| *Dasyatis centroura* (Mitchill, 1815) | Arraia-de-pedra | F; C |
| *Dasyatis say* (LeSueur, 1817) | Arraia-de-pedra | F; C |
| **Diodontidae** | | |
| *Diodon hystrix* (Linnaeus, 1758) | Baiacu-espinho; baiacu-espinheiro | F |
| *Diodon holocanthus* (Linnaeus, 1758) | Baiacu-espinho; baiacu-espinheiro | F |
| *Chilomycterus antillarum* (Jordan & Rutter, 1897) | Baiacu-espinho; baiacu-espinheiro | F |
| *Chilomycterus antennatus* (Cuvier, 1816) | Baiacu-espinho; baiacu-espinheiro | F |
| *Chilomycterus atringa* (Linnaeus, 1758) | Baiacu-espinho; baiacu-espinheiro | F |
| *Cyclichthys schoepfi* (Walbaum, 1792) | Baiacu-espinho; baiacu-espinheiro | F |
| *Chilomycterus spinosus* (Linnaeus, 1758) | Baiacu-espinho; baiacu-espinheiro | F |
| **Echeneidae** | | |
| *Echeneis naucrates* (Linnaeus, 1758) | Piolho; lebre | N |
| **Elopidae** |  |  |
| *Elops saurus* (Linnaeus, 1766) | Ubarana-espinhenta | F |
| **Engraulidae** | | |
| *Lycengraulis grossidens* (Agassiz, 1829) | Arenque; arem; erem | F; C |
| *Anchoa spinifer* (Valenciennes in Cuvier & Valenciennes, 1848) | Arenque; arem; erem | F; C |
| *Anchoa hepsetus* (Linnaeus, 1758) | Arenque; arem; erem | F; C |
| *Anchovia clupeoides* (Swainson, 1839) | Arenque; arem; erem | F; C |
| **Ephippidae** | | |
| *Chaetodipterus faber* (Broussonet, 1782) | Parum-branco | F; C |
| **Exocoetidae** | | |
| *Cheilopogon melanurus* (Valenciennes, 1847) | Avuador-de-casco | N |
| *Exocoetus volitans* (Linnaeus, 1758) | Avuador-de-casco | N |
| *Cheilopogon cyanopterus* (Valenciennes, 1847) | Avuador-de-casco | N |
| **Fistulariidae** | | |
| Fistularia tabacaria (Linnaeus, 1758) | Trombeta | N |
| **Gempylidae** | | |
| *Gempylus serpens* (Cuvier, 1829) | Espada | F; C |
| **Gerreidae** | | |
| *Eugerres brasilianus* (Cuvier, 1830) | Carapeba | F; C |
| *Diapterus auratus* (Ranzani, 1842) | Carapeba | F; C |
| *Diapterus rhombeus* (Cuvier, 1829) | Carapeba | F; C |
| *Eucinostomus argenteus* (Baird & Girard, 1854) | Carapicu | F; C |
| *Eucinostomus gula* (Quoy & Gaimard, 1824) | Carapicu | F; C |
| *Eucinostomus havana* (Nichols, 1912) | Carapicu | F; C |
| *Eucinostomus melanopterus* (Bleeker, 1863) | Carapicu | F; C |
| **Ginglymostomatidae** | | |
| *Ginglymostoma cirratum* (Bonnaterre, 1788) | Tubarão-lixa | F |
| **Gymnuridae** | | |
| *Gymnura micrura* (Bloch & Schneider, 1801) | Arraia-jamanta | F |
| *Gymnura altavela* (Linnaeus, 1758) | Arraia-lisa | F |
| **Haemulidae** | | |
| *Haemulon plumierii* (Lacepède, 1801) | Biquara; biguara | F; C |
| *Haemulon parra* (Desmarest, 1823) | Biquara; biguara | F; C |
| *Haemulon steindachneri* (Jordan & Gilbert, 1882) | Biquara; biguara | F; C |
| *Orthopristis ruber* (Cuvier, 1830) | Canguite; canguito; quanguite | F; C |
| *Anisotremus virginicus* (Linnaeus, 1758) | Carro-de-boi; boi-de-carro | F; C |
| *Pomadasys corvinaeformis* (Steindachner, 1868) | Coró-branco | F; C |
| *Conodon nobilis* (Linnaeus, 1758) | Coró-cardeiro | F; C |
| *Haemulon flavolineatum* (Desmarest, 1823) | Listrado | F; C |
| *Anisotremus surinamensis* (Bloch, 1791) | Pirambú | F; C |
| *Haemulon melanurum* (Linnaeus, 1758) | Sapuruna-de-listras | F; C |
| *Achirus achirus* (Linnaeus, 1758) | Sanhoá | F; C |
| *Achirus lineatus* (Linnaeus, 1758) | Sanhoá | F; C |
| *Trinectes paulistanus* (Miranda-Ribeiro, 1915) | Sanhoá | F; C |
| *Haemulon aurolineatum* (Cuvier, 1830) | Xila; xira | F; C |
| *Haemulon squamipinna* (Rocha & Rosa, 1999) | Xila; xira | F; C |
| **Hemiramphidae** | | |
| *Hemiramphus brasiliensis* (Linnaeus 1758) | Agulha-preta | F |
| *Hemiramphus balao* (Lesueur, 1821) | Agulha-preta | F |
| *Hyporhamphus unifasciatus* (Ranzani, 1842) | Agulha-branca | F |
| **Holocentridae** | | |
| *Holocentrus adscensionis* (Osbeck, 1765) | Mariquita | N |
| **Isthiophoridae** | | |
| *Istiophorus albicans* (Latreille, 1804) | Agulhão-de-vela | F; C |
| **Kyphosidae** | | |
| *Kyphosus incisor* (Cuvier, 1831) | Salema-azul | F |
| **Labridae** | | |
| *Caulolatilus chrysops* (Valenciennes, 1833) | Batata | F; C |
| *Halichoeres poeyi* (Steindachner, 1867) | Bronha | F; C |
| *Halichoeres bivittatus* (Bloch, 1791) | Bronha | F; C |
| *Halichoeres brasiliensis* (Bloch, 1791) | Burdião | F; C |
| *Halichoeres penrosei* (Starks, 1913) | Burdião | F; C |
| *Xyrichthys novacula* (Linnaeus, 1758) | Burdião | F; C |
| *Sparisoma axillare* (Steindachner, 1878) | Burdião | F; C |
| *Sparisoma frondosum* (Agassiz, 1831) | Burdião | F; C |
| *Sparisoma radians* (Valenciennes, 1840) | Burdião | F; C |
| *Sparisoma amplum* (Ranzani, 1842) | Burdião | F; C |
| *Sparisoma viride* (Bonnaterre, 1788) | Burdião | F; C |
| *Bodianus rufus* (Linnaeus, 1758) | Dourado | F; C |
| *Bodianus pulchellus* (Poey, 1860) | Piraúna | F; C |
| **Lamnidae** | | |
| *Carcharodon carcharias* (Linnaeus, 1758) | Tubarão-branco | N |
| **Lobotidae** | | |
| *Lobotes surinamensis* (Bloch, 1790) | Xacarona; xancarrona; | F |
| **Lutjanidae** | | |
| *Lutjanus synagris* (Linnaeus, 1758) | Ariacó | F; C |
| *Lutjanus cyanopterus* (Cuvier, 1828) | Caranha | F; C |
| *Lutjanus griseus* (Linnaeus, 1758) | Caranha | F; C |
| *Lutjanus apodus* (Walbaum, 1792) | Carapitanga | F; C |
| *Lutjanus analis* (Cuvier, 1828) | Cioba | F; C |
| *Lutjanus jocu* (Bloch & Schneider, 1801) | Dentão | F; C |
| *Ocyurus chrysurus* (Bloch, 1791) | Guaiúba | F; C |
| *Lutjanus purpureus* (Poey, 1876) | Pargo-cachucha; pargo-cachuchu | F; C |
| *Rhomboplites aurorubens* (Cuvier, 1829) | Pargo-cachucha; pargo-cachuchu | F; C |
| *Lutjanus buccanella* (Cuvier, 1828) | Pargo-ferreira; pargo-preto | F; C |
| *Lutjanus vivanus* (Cuvier, 1828) | Pargo-vidrado; pargo-vridado | F; C |
| **Malacanthidae** | | |
| *Malacanthus plumieri* (Bloch, 1786) | Pirá | F |
| **Megalopidae** | | |
| *Megalops atlanticus* (Valenciennes, 1847) | Camurim | F; C |
| **Mobulidae** | | |
| *Manta birostris* (Walbaum, 1792) | Arraia-de-orelha | F |
| *Mobula* sp. | Arraia-jamanta | F |
| **Monacanthidae** | | |
| *Cantherhines macrocerus* (Hollard, 1853) | Cangulo; canguro | F; C |
| *Cantherhines pullus* (Ranzani, 1842) | Cangulo; canguro | F; C |
| *Aluterus heudelotii* (Hollard, 1855) | Cangulo; canguro | F; C |
| *Aluterus monoceros* (Linnaeus, 1758*)* | Cangulo; canguro | F; C |
| *Aluterus schoepfii* (Walbaum, 1792) | Cangulo; canguro | F; C |
| *Aluterus scriptus* (Osbeck, 1765) | Cangulo; canguro | F; C |
| *Stephanolepis setifer* (Bennett, 1831) | Cangulo; canguro | F; C |
| *Monacanthus ciliatus* (Mitchill, 1818) | Cangulo; canguro | F; C |
| **Mugilidae** | | |
| *Mugil platanus* (Günther, 1880 | Saúna; coípe; tamatarana; tainha | F; C |
| *Mugil liza* (Valenciennes in Cuvier & Valenciennes, 1836) | Saúna; coípe; tamatarana; tainha | F; C |
| *Mugil curema* (Valenciennes, 1836) | Saúna; coípe; tamatarana; tainha | F; C |
| *Mugil trichodon* (Poey, 1875) | Saúna; coípe; tamatarana; tainha | F; C |
| *Mugil incilis* (Hancock, 1830) | Saúna; coípe; tamatarana; tainha | F; C |
| **Muraenidae** | | |
| *Channomuraena vittata* (Richardson, 1845) | Moréia | F |
| *Gymnothorax moringa* (Cuvier, 1829) | Moréia | F |
| *Gymnothorax vicinus* (Castelnau, 1855) | Moréia | F |
| *Gymnothorax funebris* (Ranzani, 1840) | Moréia | F |
| *Muraena pavonina* (Richardson, 1845) | Moréia | F |
| **Myliobatidae** | | |
| *Aetobatus narinari* (Euphrasen, 1790) | Arraia-pintada | N |
| **Narcinidae** | | |
| *Narcine bancrofti* (Griffith & Smith, 1834) | Cação-choque; choqueiro (raia) | N |
| *Narcine brasiliensis* (Olfers 1831) | Cação-choque; choqueiro (raia) | N |
| **Ogcocephalidae** | | |
| *Ogcocephalus vespertilio* (Linnaeus, 1758) | Peixe-morcego | N |
| **Ophichthidae** | | |
| *Myrichthys ocellatus* (Le Suer, 1825) | Muriongo | N |
| *Myrophis punctatus* (Lütken, 1852) | Muriongo | N |
| *Myrichthys breviceps* (Richardson, 1848) | Muriongo | N |
| **Ostraciidae** | | |
| *Acanthostracion quadricornis* (Linnaeus, 1758) | Baiacu-de-chifre; baiacu-vaquinha | N |
| *Acanthostracion polygonius* (Poey, 1876) | Baiacu-de-chifre; baiacu-vaquinha | N |
| **Paralichthyidae** | | |
| *Citharichthys macrops* (Dresel, 1885) | Sóia; solha | N |
| *Citharithys spilopterus* (Günther, 1862) | Sóia; solha | N |
| *Etropus crossotus* (Jordan & Gilbert, 1882) | Sóia; solha | N |
| *Paralichthys brasiliensis (Ranzani, 1842)* | Sóia; solha | N |
| *Syacium micrurum* (Ranzani, 1842) | Sóia; solha | N |
| *Syacium papillosum* (Linnaeus, 1758) | Sóia; solha | N |
| **Polynemidae** | | |
| *Polydactylus virginicus* (Linnaeus, 1758) | Barbado; barbudo | F |
| **Pomacanthidae** | | |
| *Holacanthus ciliaris* (Linnaeus, 1758) | Parum-amarelo | F; C |
| *Holacanthus tricolor* (Bloch, 1795) | Parum-amarelo | F; C |
| *Pomacanthus arcuatus* (Linnaeus, 1758) | Parum-listrado | F; C |
| *Epinephelus adscensionis* (Osbeck, 1765) | Parum-listrado | F; C |
| *Pomacanthus paru* (Bloch, 1787) | Parum-preto | F; C |
| **Priacanthidae** | | |
| *Selar crumenophthalmus* (Bloch, 1793) | Garapau | F |
| *Heteropriacanthus cruentatus* (Lacepède, 1801) | Oiam; olhão | F |
| *Pristigenys alta* (Gill, 1862) | Oiam; olhão | F |
| *Priacanthus arenatus* (Cuvier, 1829) | Oiam; olhão | F |
| *Decapterus punctatus* (Cuvier, 1829) | Oiam; olhão | F |
| **Pristigasteridae** | | |
| *Pellona harroweri* (Fowler, 1917) | Sardinha | F |
| *Pellona flavipinnisi* (Valenciennes, 1836) | Sardinha | F |
| *Opisthonema oglinum* (Le Sueur, 1818) | Sardinha | F |
| **Rachycentridae** | | |
| *Rachycentron canadum* (Linnaeus, 1766) | Beijupirá; bijupirá; cação-de-escama | F; C |
| **Rhicodontidae** | | |
| *Rhincodon typus* (Smith, 1828) | Tubarão-baleia; Tubarão-pintado | N |
| **Rhinobatidae** | | |
| *Rhinobatos percellens* (Walbaum, 1792) | Cação-viola | N |
| *Rhinobatos lentiginosus* (Lesaa *et al*., 1995; Felix 1998) | Cação-viola | N |
| **Rhinopteridae** | | |
| *Rhinoptera bonasus* (Mitchill, 1815) | Arraia-boca-de-gaveta; Arraia-mão-de-tranca | F |
| **Scaridae** | | |
| *Scarus zelindae* (Moura, Figueiredo & Sazima, 2001) | Burdião | F |
| **Sciaenidae** | | |
| *Larimus breviceps* (Cuvier, 1830) | Boca-mole | F; C |
| *Stellifer microps* (Steindachner, 1864) | Cabeça-dura | F; C |
| *Stellifer naso* (Jordan, 1889) | Cabeça-dura | F; C |
| *Stellifer rastrifer* (Jordan, 1889) | Cabeça-dura | F; C |
| *Paralonchurus brasiliensis* (Steidachner, 1875) | Cabeça-dura | F; C |
| *Odontoscion dentex* (Cuvier, 1830) | Cabeça-dura | F; C |
| *Micropogonias furnieri* (Desmarest, 1823) | Cururuca; curuca | F; C |
| *Micropogonias undulatus* (Linnaeus, 1766) | Cururuca; curuca | F; C |
| *Menticirrhus americanus* (Linnaeus, 1758) | Judeu | F; C |
| *Menticirrhus littoralis* (Holbrook, 1855) | Judeu | F; C |
| *Cynoscion leiarchus* (Cuvier, 1830) | Pescada-branca | F; C |
| *Macrodon ancylodon* (Bloch & Schneider, 1801) | Pescada-curuvina | F; C |
| *Cynoscion acoupa* (Lacepède, 1801) | Pescada-de-dente | F; C |
| *Cynoscion microlepidotus* (Cuvier, 1830) | Pescada-de-dente | F; C |
| *Cynoscion virescens* (Cuvier, 1830) | Pescada-de-dente | F; C |
| *Cynoscion* sp. | Pescada-amarela | F; C |
| *Cynoscion* sp. | Pescada-bico-fino | F; C |
| *Cynoscion* sp. | Pescada-ticupá | F; C |
| **Scombridae** | | |
| *Euthynnus alletteratus (Rafinesque, 1810)* | Alvacora; albacora | F; C |
| *Thunnus alalunga* (Bonaterre, 1788 ) | Alvacora; albacora | F; C |
| *Thunnus albacares* (Bonaterre, 1788 ) | Alvacora; albacora | F; C |
| *Thunnus atlanticus* (Lesson, 1839) | Alvacora; albacora | F; C |
| *Thunnus obesus* (Lowe, 1839) | Bonito | F; C |
| *Katsuwonus pelamis* (Linnaeus, 1758) | Bonito | F; C |
| *Auxis thazard* (Lacépède, 1803 ) | Bonito | F; C |
| *Auxis thazard brachydorax* (Collette & Aadland, 1996) | Bonito | F; C |
| *Aconthocybium solandri* (Cuvier, 1832) | Cavala | F; C |
| *Scomberomorus cavalla* (Cuvier, 1829) | Serra | F; C |
| *Scomberomorus brasiliensis* (Collette, russo & Zavala-Camin, 1978) | Serra | F; C |
| *Scomberomorus regalis* (Bloch, 1793) | Serra | F; C |
| **Scorpaenidae** | | |
| *Scorpaena plumieri* (Bloch, 1789) | Aniquim; anequim | N |
| **Serranidae** | | |
| *Epinephelus adscensionis* (Osbeck, 1765) | Gato | F; C |
| *Epinephelus niveatus* (Valenciennes, 1828) | Garoupa; garopa | F; C |
| *Epinephelus marginatus* (Lowe, 1834) | Garoupa; garopa | F; C |
| *Epinephelus morio* (Valenciennes, 1828) | Guaiúba | F; C |
| *Paranthias fucifer* (Valenciennes, 1828) | Jacundá | F; C |
| *Diplectrum radiale* (Quoy & Gaimard, 1824) | Jacundá | F; C |
| *Diplectrum formosum* (Linnaeus, 1766) | Jacundá | F; C |
| *Epinephelus itajara* (Lichtenstein, 1822) | Mero | F; C |
| *Myripristis jacobus* (Cuvier, 1829) | Oiuda | F; C |
| *Etelis oculatus* (Valenciennes, 1828) | Pargo-piranga | F; C |
| *Cephalopholis fulva* (Linnaeus, 1758) | Piraúna | F; C |
| *Rypticus randalli* (Courtenay, 1967) | Sabão | F; C |
| *Rypticus bistrispinus* (Mitchill, 1818) | Sabão | F; C |
| *Rypticus saponaceus* (Bloch & Schneider, 1801) | Sabão | F; C |
| *Serranus flaviventris* (Cuvier, 1829) | Sapé | F; C |
| *Paralabrax dewegeri* (Metzelaar, 1919) | Serigado; sirigado | F; C |
| *Mycteroperca bonaci* (Poey, 1860) | Serigado; sirigado | F; C |
| **Sparidae** | | |
| *Calamus pennatula* (Guichenot, 1868) | Pena | F; C |
| *Calamus calamus* (Valenciennes, 1830) | Pena | F; C |
| *Calamus penna* (Valenciennes, 1830) | Pena | F; C |
| *Pagrus pagrus* (Linnaeus, 1758) | Salema | F; C |
| *Archosargus rhomboidalis* (Linnaeus, 1758) | Sargo | F; C |
| *Archosargus probatocephalus* (Walbaum, 1792) | Sargo | F; C |
| **Sphyraenidae** | | |
| *Sphyraena barracuda* (Walbaum, 1792) | Barracuda | F; C |
| *Sphyraena guachancho* (Cuvier, 1829) | Bicuda | F; C |
| *Sphyraena picudilla* (Poey, 1860) | Bicuda | F; C |
| **Sphyrnidae** | | |
| *Sphyrna tiburo* (Linnaeus, 1758) | Cação-panã; tubarão-cornuda; martelo; tubarão-cabeça-de-martelo; tintureira | N |
| *Sphyrna lewini* (Griffith & Smith, 1834) | Cação-panã; tubarão-cornuda; martelo; tubarão-cabeça-de-martelo; tintureira | N |
| *Sphyrna zygaena* (Linnaeus, 1758) | Cação-panã; tubarão-cornuda; martelo; tubarão-cabeça-de-martelo; tintureira | N |
| **Stromateidae** | | |
| *Peprilus paru* (Linnaeus, 1758) | Mocinha | F |
| **Syngnathidae** | | |
| *Hippocampus reidi* (Ginsburg, 1933) | Cavalo-marinho | O |
| *Hippocampus aff. erectus* (Perry, 1810) | Cavalo-marinho | O |
| **Synodontidae** | | |
| *Synodus foetens* (Linnaeus, 1766) | Traíra | F |
| *Synodus intermedius* (Spix & Agassiz, 1829) | Traíra | F |
| *Trachinocephalus myops* (Forster, 1801) | Traíra | F |
| **Tetraodontidae** | | |
| *Lactophrys trigonus* (Linnaeus, 1758) | Baiacu-araldo | F |
| *Lactophrys* sp. | Baiacu-carlitão | F |
| *Lagocephalus laevigatus* (Linnaeus, 1766) | Baiacu-guarajuba | F |
| *Sphoeroides dorsalis* (Longley, 1934) | Baiacu-guarajuba | F |
| *Sphoeroides spengleri* (Bloch, 1785) | Baiacu-pintado | F |
| *Sphoeroides tyleri* (Shipp, 1972) | Baiacu-pintado | F |
| *Sphoeroides greeleyi* (Gilbert, 1900) | Baiacu-pintado | F |
| *Sphoeroides testudineus* (Linnaeus, 1758) | Baiacu-pintado | F |
| **Triakidae** | | |
| *Mustelus canis* (Mitchill, 1815 ) | Cação-toalha | F |
| *Squalus cubensis* (Howell Rivero, 1936) | Cação-toalha | F |
| **Trichiuridae** | | |
| *Trichiurus lepturus* (Linnaeus, 1758) | Espada | F; C |
| **Xiphiidae** | | |
| *Xiphias gladius* (Linnaeus, 1758) | Agulhão-de-vela | F; C |

**Legend:** F - Food resource, C – Commercial, M- Medicinal, O- Ornamentation and decoration, N - not used.
